# Supplementary material for: Causal Relationship between Aging and Anorexia Nervosa: A White-Matter-Microstructure-Mediated Mendelian Randomization Analysis
Source: Biomedicines. 2024 Aug 16;12(8):1874. doi: 10.3390/biomedicines12081874 (PMC11351342; doi:10.3390/biomedicines12081874)
Supplement: Supplementary file 1 [file biomedicines-12-01874-s001.zip › Supplementary Figure/Figure S4/Singe SNP plot of MR analysis for TL to ubm-b-1876.pdf]

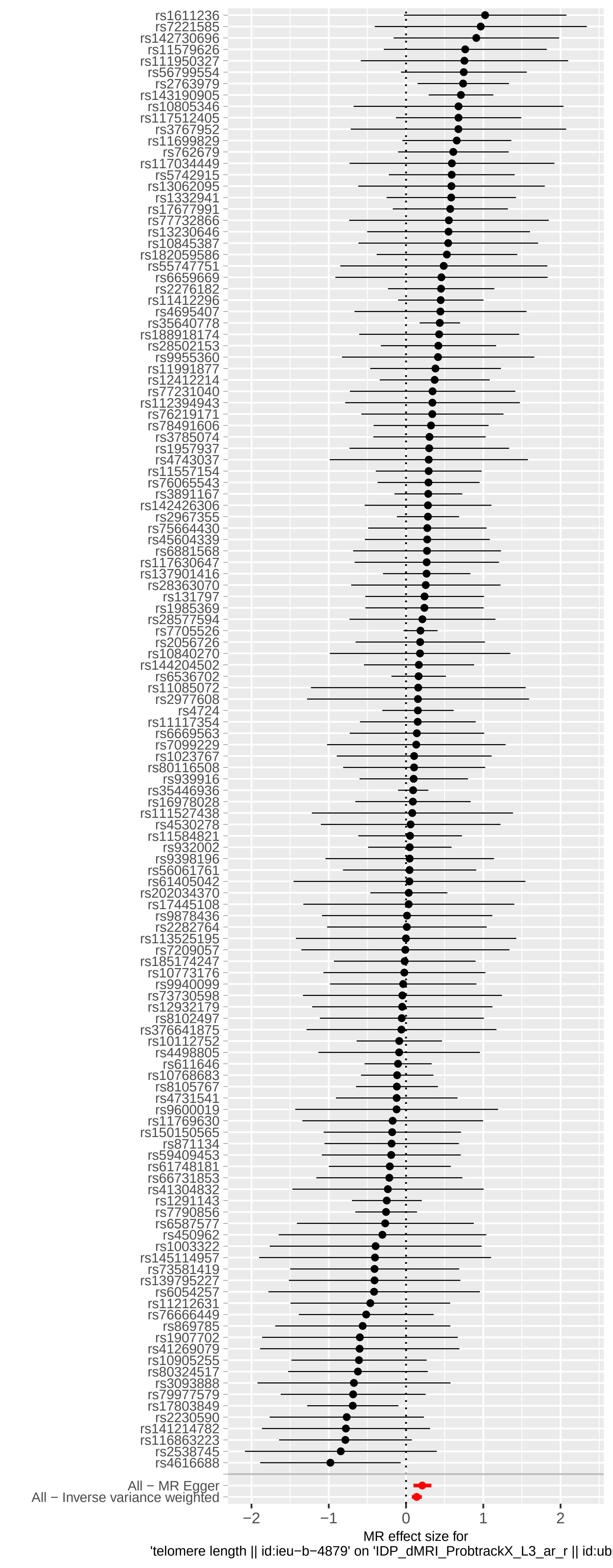
rs1611236
rs7221585
rs142730696
rs11579626
rs111950327
rs56799554
rs2763979
rs143190905
rs10805346
rs117512405
rs3767952
rs11699829
rs762679
rs117034449
rs5742915
rs13062095
rs1332941
rs17677991
rs77732866
rs13230646
rs10845387
rs182059586
rs55747751
rs6659669
rs2276182
rs11412296
rs4695407
rs35640778
rs188918174
rs28502153
rs9955360
rs11991877
rs12412214
rs77231040
rs112394943
rs76219171
rs78491606
rs3785074
rs1957937
rs4743037
rs11557154
rs76065543
rs3891167
rs142426306
rs2967355
rs75664430
rs45604339
rs6881568
rs117630647
rs137901416
rs28363070
rs131797
rs1985369
rs28577594
rs7705526
rs2056726
rs10840270
rs144204502
rs6536702
rs11085072
rs2977608
rs4724
rs11117354
rs6669563
rs7099229
rs1023767
rs80116508
rs939916
rs35446936
rs16978028
rs111527438
rs4530278
rs11584821
rs932002
rs9398196
rs56061761
rs61405042
rs202034370
rs17445108
rs9878436
rs2282764
rs113525195
rs7209057
rs185174247
rs10773176
rs9940099
rs73730598
rs12932179
rs8102497
rs376641875
rs10112752
rs4498805
rs611646
rs10768683
rs8105767
rs4731541
rs9600019
rs11769630
rs150150565
rs871134
rs59409453
rs61748181
rs66731853
rs41304832
rs1291143
rs7790856
rs6587577
rs450962
rs1003322
rs145114957
rs73581419
rs139795227
rs6054257
rs11212631
rs76666449
rs869785
rs1907702
rs41269079
rs10905255
rs80324517
rs3093888
rs79977579
rs17803849
rs2230590
rs141214782
rs116863223
rs2538745
rs4616688
All − MR Egger
All − Inverse variance weighted
−2
−1
0
1
2
MR effect size for
'telomere length || id:ieu−b−4879' on 'IDP_dMRI_ProbtrackX_L3_ar_r || id:ub
